# Medicinal plants of Otwal and Ngai Sub Counties in Oyam District, Northern Uganda

**DOI:** 10.1186/1746-4269-7-7

**Published:** 2011-01-17

**Authors:** Maud M Kamatenesi, Annabel Acipa, Hannington Oryem-Origa

**Affiliations:** 1 Department of Botany, Makerere University, P.O Box 7062, Kampala, Uganda; 2 Institute of Environment and Natural Resources Makerere University, P.O Box 7062, Kampala, Uganda

## Abstract

**Background:**

An ethnobotanical study was carried out in four parishes in the Ngai and Otwal Sub Counties in Oyam district, Northern Uganda, where insurgency has been prevalent for the past 20 years. Documenting medicinal plant species used in treating various health conditions among the local people.

**Methods:**

Information was obtained from mainly the local population, the traditional healers and other experienced persons through interviews, formal and informal discussions and field excursions.

**Results:**

Seventy one plant species were reported for use in the treatment of various diseases in the study area. These plant species belongs to 41 families, with Asteraceae being the most represented. Roots were ranked the commonest plant part used. Oral administration was the most frequently used route of administration. A total of 41 different health conditions were reported to be treated by use of medicinal plant species. Thirty nine percent of the recorded plant species were reported for treating stomach related ailments.

**Conclusion:**

The use of medicinal plants in primary healthcare is still a common practice in Ngai and Otwal Sub Counties. The trust they have is built on the curative outcome properties claimed, poverty and armed conflict that lead to inadequate healthcare facilities. The generation gap caused by the over 20 years of insurgency in the area has brought about knowledge gap on the usage of medicinal plant species between the young and the older generation.

## Background

World wide over 80% of the people depend on medicinal plant species to meet their day today healthcare needs [1]. Rural household of Uganda rely heavily on plant resources for food, fodder and herbal medicine [2]. Tabuti [2] further asserted that savanna environment contains many plant resources of economic values such as foods and medicines. These resources are widely relied on by rural communities in developing countries because of inefficiencies in service delivery or because social services and goods are unaffordable. For this reason many people are currently resorting to traditional medicine for primary health care due to high costs in accessibility, cultural compatibility, self-reliance among others [3]. They also employ herbal medicines because of cultural preferences and perceived effectiveness [4,5].

Medicinal plant species form a main part of treatment for the rural poor. Traditional medicine usage in rural Ugandan population for day-to-day health care needs is close to 90% [6]. Kamatenesi and Oryem [6] further reported that women and children form the bulk of the people reliant on herbal medicine. According to Katuura et al [7], malaria was reported to be the most common condition treated by traditional healers in Mbarara District. The use of traditional herbal remedies is encountered in both rural and urban areas in Mali and that traditional medicine is one of the surest means to achieve total health care coverage for African's population [8].

Discourses on the future of traditional medicine in Africa and other indigenous societies often assume government recognition and integration into the formal health care systems [9].

In certain areas in Nigeria, the only health care providers close to the people are the traditional medical practitioners [10]. However, it should be noted that medicinal plant species have also been discovered to have other uses as some could be used as vegetables, fruits, trees and ornamentals [11].

Health services in Oyam District are inadequate, and only 15 out of the 43 parishes in Oyam District have health facilities. Maternal mortality rate is still high because clean and safe deliveries are at only 14% because it is mainly the traditional birth attendants (TBA) who play a significant role [12].

## Methods

### Study area

**Figure 1:**
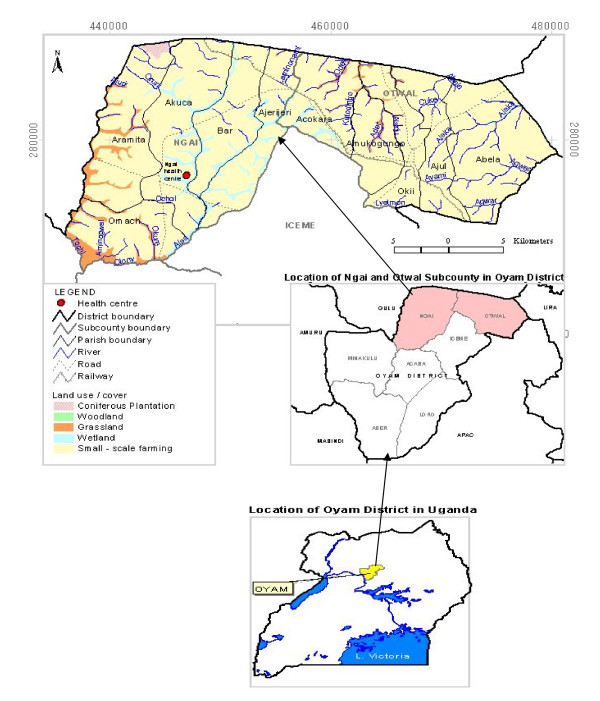
**Location of Ngai and Otwal Sub Counties in Oyam District in Northern Uganda**.

This study was carried out in Ngai and Otwal sub counties in Oyam District which is situated in northern Uganda on coordinates 02°14'N 32°23'E (Figure 1) [13]. The sampling sites were located in the Parishes of Aramita, Akuca and Omac from Ngai Sub County and Abela from Otwal Sub-County. The study was conducted between August 2007 and February 2008 in Oyam District, Northern Uganda.

### Data collection

Ethnobotanical information was obtained through informed consent semi-structured interviews with key informants. The key informants consisted of health workers, renowned herbalists, and local leaders. However, the bulk of the respondents were local residents who were identified through household numbers. Knowledge on the use of medicinal plant species was documented, the local name of plant species, diseases or ailments they treat, part of plant used, methods of preparation and administration were recorded.

In addition, a total of 84 households were interviewed using questionnaires, after being randomly chosen from the total household list from the LC I (Local Councilor One) chairperson. Forty four households from Ngai and another 40 from Otwal Sub Counties were interviewed through the use of questionnaire. Some questions asked included; village of respondent, level of education, knowledge on medicinal plant species among others.

For more studies and information, three focus group discussions were conducted in Acandano village in Ngai Sub-County and Abela primary school and Ojwi centre in Otwal Sub-County. In this case the respondents were asked research guided questions. The groups comprised of children 15, women 20 and men 12. The groups participated voluntarily at the invitation of LC 1 chairman. The focus group discussion helped discover the extent of distribution of knowledge on medicinal plant species.

### Voucher Specimens and Sample Collection

Voucher specimens of the documented plant species were collected according to standard practice, including roots, flowers, and fruits where possible [14]. Collection only involved samples that were identified by the respondent. The voucher specimens were delivered to Makerere University Botany Herbarium where further identification and classification was done. Scientific names of plant species were identified based on International Plant Name Index (IPNI: http://www.ipni.org).

## Results

A total of 110 respondents were interviewed from the study area; 46 were females and males were 64 as shown in table 1.

**Table 1:** Total number of respondents that were interviewed in the study area

**Respondents**			**Total**
Males	Females		
64 (58%)	46 (42%)		110
**Age Characteristics of Respondents**			
**13-24 years**	**25-37 years**	**38-49 years**	**50 years and above**
17 (15%)	32 (29%)	27 (25%)	34 (31%)

From the research findings, 71 medicinal plant species both wild and cultivated belonging to 42 families were documented and identified in the study area (Table 2). The family Asteraceae (5 species) was the most represented followed by Leguminosae and Lamiaceae (4 species) plant species each; Solanaceae, Poaceae, Eurphorbiaceae, and Zingiberaceae had 3 plant species in each family, and the remaining families had two and one species. With regard to growth habits, the plant species consisted of shrubs (39%), herbs and climbers (36.6%), trees (21%) and grasses (4%).

**Table 2:** Medicinal plants their habits, growth habit, frequency of mention, plant part used, diseases treated, methods of preparation and administration.

Family	Taxon	Habitat	Habit	Plant part used	Disease	Number of diseases treated	Freq of mention of plant	Methods of Preparation	Administration
Amaranthaceae	*Pupalia lappacea *Juss. AA-49-07	Wooded grassland	SH	R	Syphilis	1	2	Crushed, boiled*	Extract drunk

Anacardiaceae	*Mangifera indica *L. AA-53-07	Homestead	T	B	Diarrhoea	2	8	Crushed, mixed in cold water	Extract drunk twice a day
							
				R	Cough			Crushed, mixed in cold water	Extract drunk

Apocynaceae	*Carissa edulis *(Forssk) Vahl. AA-59-07	Grassland	SH	R	Epilepsy	2	3	Crushed, mixed in cold water	Extract drink
							
				S	Abdominal pain			Crushed, mixed in cold water	Extract drunk

Asclepiadaceae	*Mondia whiteii *Skeels AA-57-07	Forest	C	R	Flu, cold	4	8	Crushed , mixed in cold water	Extract drunk
							
				R	Abdominal pain			Crushed, mixed in cold water	Extract drunk twice a day
							
				R	Headache, cough			Picked, cleaned	Chewed

Asparagaceae	*Asparagus africanus *Hochst. ex.A. Rich AA-48-07	Open grassland	SH	R	Swollen body	1	4	Crushed, mixed in cold water	Extract drunk one glass twice a day , rub on skin cuts

Asteraceae	*Acmela canlirhiza *Delile AA-64-07	Garden edge, road side	H	R, L	Cough	2	2	Dried, powdered	Extract drunk three teaspoon twice a day
							
				R	Retained placenta			Crushed, mixed in warm water*	Extract drunk
	
	*Biden pilosa *L. AA-47-07	Garden	H	L	Wounds	1	3	Dried, powdered	Applied on wound
	
	*Echinops amplexicaulis *Oliv. AA-07-07	Open grassland	SH	R	Hydrocelle	7	7	Crushed, mixed in cold water	Extract drunk three times a day
							
				R	Hernia scrotal			Crushed, mixed in cold water*	Extract drunk
							
				R	Stomachache			Crushed , boiled	Extract drunk 200 ml once a day
							
				R	TB			Crushed, boiled	Extract drunk quarter glass for adults twice a day, two spoonful twice a day for children
							
				R	snake bite, whooping cough, syphilis			Crushed, mixed in cold water	Extract drunk one glass twice a day
	
	*Conyza sumatrensis *(Retz.) E.Walker AA-35-07	Open grassland	SH	L	Wounds	3	12	Crushed	Juice onto fresh wound
							
				L	Sore throat			Picked, cleaned	Chewed, juice swallowed three times a day
							
				L	Ring worm			Crushed	Extract rubbed on affected part once a day
	
	*Aspilia africana *C.D Adams AA-37-07	Open grassland Abandoned gardens, road side	SH	R	Sore throat	8	3	Crushed, mixed in cold water	Extract drunk
							
				R	Diarrhoea, dysentery			Crushed, mixed in cold water	Extract drunk quarter a glass three times a day
							
				R	Body cleanser			Crushed, mixed in cold water	Extract drunk
							
				R	Antidote			Crushed, mixed in cold water	Extract drunk
							
				R	Wounds			Crushed	Juice squeezed onto wound
							
				R	Induce appetite			Picked, cleaned	Chewed, juice swallowed
							
				R	snake bite			Crushed, mixed in cold water	Extract drunk
	
	*Microglossa pyrifolia *(Cam) O. Ktze AA-36-07	Wooded grassland	SH	R	Anti venom	2	2	Crushed	Rubbed on skin cuts
							
				L	Epilepsy			Crushed, added in bath water	Used for bathing, burnt in patient room

	*Vernonia amygdalina *Del. AA-46-07	Open grassland	SH	R	Cough	9	10	Crushed, mixed in cold water	Extract drunk
							
				R	Abdominal pain			Crushed, mixed in cold water	Extract drunk twice a day
							
				L	Wound			Crushed	Extract applied on wound
							
				L	Malaria			Crushed, mixed in cold water	Extract drunk
							
				R	Swollen stomach			Crushed, mixed in cold water	Extract drunk
							
				R	Hernia			Dried, powdered	Extract drunk 10 ml twice a day, extract rubbed on skin cuts
							
				R	Headache			Crushed, mixed in cold water	Extract drunk 2 spoonful thrice a day
							
				R	STI			Crushed, mixed in cold water	Extract drunk 500 ml thrice a day
							
				R	Diarrhoea			Crushed, mixed in warm water	Extract drunk 500 ml once a day

	Vernonia sp. AA-02-07	Open grassland, garden	H	R, L	Backbone disease	1	3	Crushed, boiled , Heated over fire	Extract drunk , heated leaves massage body twice a day

Bignoniaceae	*Markhamia platycalyx *Sprague AA-54-07	Wooded grassland	T	R	Ease child bearing, Induces labour	1	1	Crushed, mixed in warm water	Extract drunk one glass once a day
	
	*Stereospermum kunthianum *Cham. AA-55-07	Wooded grassland	T	R	Wounds	1	1	Dried, powdered	Applied on wound
	
	*Kigelia africana *(Lam.) Benth AA-60-07	Wooded grassland	T	L	Eye disease	3	2	Crushed	Squeezed in eye
							
				B	Poison antidote			Crushed, boiled	Extract drunk once a day
							
				S	Impotence			Dried, powdered	Extract drunk, eaten.

Caesalpiniaceae	*Cassia siamea *Lam. AA-56-07	Semi cultivated	T	R	Sore throat	2	4	Crushed and mixed in cold water	Extract drunk
							
				L	Abdominal pain			Picked, cleaned	Chewed, liquid swallowed

Capparaceae	*Cleome gynandra *L.AA-61-07	Homestead, garden	H	L	Headache	3	5	Crushed	Rubbed on forehead
							
				L	Ring worm			Crushed	Rubbed on affected area
							
				R	Eye disease			Crushed	Dropped in eye

Caricaceae	*Carica papaya *L. AA-43-07	Homestead	T	R	body pain by witch craft	1	3	Crushed	Rubbed on body twice a day

Celastraceae	*Maytenus senegalensis *(Lam) Exell AA-45-07	Forest	T	R	Epilepsy	2	1	Crushed, mixed in cold water*	Extract drunk 50 ml three times a day
							
				R	Miscarriage			Crushed, mixed in cold water	Extract drunk 300 ml two times a day

Chenopodiaceae	*Chenopodium ambrosioides *L. AA-50-07	Around home stead	H	L	Headache	2	4	Crushed, mixed in hot water	Steam inhaled, heated leaves placed on face
							
				L	Epilepsy			Crushed, mixed in cold water	Extract drunk 25 ml twice a day, applied on skin cuts

Combretaceae	*Combretum molle *R.Br.G. Don AA-44-07	Swampy area, forest edge	T	R	Cough	1	1	Dried, powdered added into one glass of water	Drunk twice a day
	
	*Combretum collinum *Fresen AA-42-07	Open grassland	T	L	Cough	4	12	Crushed, mixed in cold water*	Extract drunk twice a day
							
				R, B	Wounds			Crushed	Juice squeezed on wound
							
				R, B	Diarrhoea,			Crushed, mixed in cold water	Extract drunk 4 teaspoon twice a day
							
				R, B	Abdominal pain			Crushed, mixed in cold water	

Cucurbitaceae	*Cucurbita maxima *Wall. AA-38-07	Gardens, antihill	C	R	Abdominal pain	1	1	Crushed, mixed in cold water	Extract drunk
	
	*Momordica foetida *Schum. AA-52-07	Antihill	C	R	STI	3	2	Crushed, mixed in cold water	Extract drunk one glass once a day
							
				R	Cough, abdominal pain			Crushed, mixed in cold water	Extract drunk one glass twice a day
	
	*Kedrostis foetidissima *Cogn. AA-41-07	Open grassland	C	R	Measles	1	1	Crushed, mixed in cold water	Extract drunk once a day

Dioscoreaceae	*Dioscorea *sp AA-62-07	Garden	H	L	Loss of appetite	1	1	Crushed, boiled	Eaten

Eurphorbiaceae	*Euphorbia hirta *L. AA-71-07	Garden, along roadside	H	R	Cough	2	6	Crushed, mixed in cold water	Extract drunk three times a day
							
				St	Fresh wound			Sap collected	Applied on wound two times a day
	
	*Fluggae virosa *(Willd.) Voigt AA-40-07	Wooded grassland	SH	R	Miscarriage	1	2	Crushed, mixed in cold water	Extract drunk 250 ml twice a day

Fabaceae	*Piliostigma thonningii *(Schumach.) Milne-Redh. AA-44-07	Open grassland	T	L	STI	2	6	Crushed, mixed in cold water	Extract drunk 750 ml thrice a day
							
				St	Diarrhoea			Crushed, mixed in warm water	Drink one teaspoon a day
	
	*Cassia nigricans *Vahl. AA-31-07	Open grassland	SH	St	Wound	3	2	Crushed	Apply on skin cuts
							
				L	Worms			Crushed, mixed in cold water	Extract drunk
							
				L	Stomachache			Crushed	Smear on stomach
	
	*Erythrina abyssinica *Lam. AA-29-07	Grassland	T	R	Toothache	1	2	Crushed, boiled	Massage tooth

Labiatae	*Hoslundia opposita *Vahl. AA-09-07	Open grass land	H	R	Epilepsy	2	6	Crushed, mixed in cold water	Extract drunk two times a day, applied as nasal drop.
							
				R	Whole body swelling			Crushed, boiled	Extract drunk

Lamiaceae	*Clerodendrum myricoides *R.Br. & Vatke AA-30-07	Open grassland	S	R	Body pains	2	4	Crushed	Rub on skin cuts
							
				R	Cataracts			Crushed	Extract dropped in eye twice a day
	
	*Ocimum basilicum *L. AA-32-07	Compound edge	H	L	Eye cataract	3	3	Crushed	Extract squeezed, dropped in eye twice a day
							
				L	Fever			Crushed, mixed in warm water	Massage body, add in bathing water
							
				L	Malaria			Crushed, mixed in warm water	Extract drunk
	
	*Vitex doniana *Sweet AA-25-07	Wooded grassland	T	R	Eye disease	1	1	Crushed, mixed in cold water	Extract dropped in eye

Leguminosae	*Rhynchosia densiflora *Wall. AA-27-07	Wooded grassland	SH	R	Dysentery	1	8	Crushed, mixed in cold water	Extract drunk two teaspoon twice a day
	
	*Indigofera arrecta *Hochst.ex. A. Rich AA-26-07	Open garden	SH	L	Body swelling	4	5	Crushed	Rubbed on skin
							
				R	Round worms			Crushed, mixed in warm water	Extract drunk 200 ml once a day
							
				R	Headache			Crushed, mixed in cold water	Extract drunk
							
				R	Sore throat			Crushed, mixed in cold water	Extract drunk twice a day
	
	*Acacia hockii *De Wild AA-24-07	Open grassland	T	R	Malaria + cough	1	1	Crushed, mixed in cold water	Extract drunk two times a day
	
	*Acacia sieberiana *Tausch AA-23-07	Wooded grassland	T	R	Epilepsy	2	1	Crushed, mixed in cold water	Extract drunk
							
				R	Dysentery			Crushed, mixed in cold water*	Extract drunk half a Aglass two times a day

Loganiaceae	*Strychnos innocua *Delile. AA-12-07	Swamps	T	R	Witchcraft	1	1	Crushed, mixed in cold water	Extract sprinkled on patient

Meliaceae	*Trichilia capensis *Pers. AA-22-07	Grassland,	SH	R	Stomachache	8	6	Crushed , mixed in cold water	Extract drunk 50 ml once a day
							
				R	Stops miscarriage			Crushed , mixed in cold water	Extract drunk half glass twice a day
							
				R	West pain			Dried , powdered	Powder added in water making 10 ml , drunk two times a day
							
				R	Urine pain			Crushed , mixed in cold water	Extract drunk two times a day
							
				R	Back ache after birth			Crushed , mixed in cold water	Extract drunk
							
				R	Worms			Crushed, mixed in water	Extract drunk
							
				R	Diarrhoea, cough			Crushed , boiled	Extract drunk 200 ml once a day
	
	*Trichilia emetica *Vahl. AA-21-07	Open grassland	H	R	Snake bite	3	11	Crushed , mixed in cold water	Extract drunk, crushed leaves rubbed on skin cuts
							
				R	Stomachache			Crushed , mixed in cold water	Extract drunk once a day
							
				R	prevent poison			Crushed , mixed in cold water	Extract drunk
							

Menispermaceae	*Cissampelos mucronata *A.Rich. AA-33-07	Garden edges	H	R	Abdominal pain	1	1	Crushed , mixed in cold water	Extract drunk three times a day

Mimosaceae	*Albizia coriaria *Welw. AA-58-07	Wooded grassland	T	B	Diarrhoea	1	1	Crushed , mixed in cold water	Extract drunk

Moraceae	*Ficus vallis *Chaude AA-20-07	Wooded grassland	T	R	Dysentery, diarrhea	3	7	Crushed , mixed in cold water	Extract drunk half glass once a day
							
				B	Ring worm			Sap collected	Smeared on affected area twice a day

Musaceae	*Musa *spp AA-69-07	Garden	T	F	Diarrhoea	3	1	Sap collected	Sap drunk thrice a day
							
				Fl	Wound			Crushed , mixed in cold water	Extract applied on wound
							
				B	Ring worm			Crushed	Smear on affected area once a day

Myrtaceae	*Eucalyptus globulus *Labill. AA-68-07	Home stead	T	L	Cough	1	5	Crushed , boiled	Extract drunk four teaspoon twice a day

Papilionaceae	*Crotalaria ochroleuca* G.Don AA-04-07	Garden	SH	L	Stomachache	1	1	Crushed , boiled	Eaten
	
	*Cajanus cajan *(L.) Druce AA-17-07	Garden	SH	L	Malaria	1	1	Crushed , mixed in cold water	Extract drunk 100 ml once a day

Poaceae	*Imperata cylindra *P.Beauv. AA-67-07	Open grassland	G	R	Abdominal pain	1	1	Crushed , mixed in cold water	Extract drunk
	
	*Pennisetum trachyphyllum *Pilg. AA-66-07	Garden, dry land	G	R	Abdominal pain	1	1	Crushed , mixed in cold water	Extract drunk
	
	*Sporobulus africanus *(Poir.) Roebyns AA-65-07	Open grassland	G	R	Retained placenta	1	2	Crushed , mixed in cold water	Extract drunk500 ml once a day

Polygolaceae	*Securidaca longipedunculata *Fres. AA-19-07	Open grassland	T	R	Body pains,	4	19	Crushed	Rubbed on skin cuts once a day
							
				R	Headache			Crushed	Rubbed on skin cuts once a day
							
				R	Skin disease			Crushed , mixed in cold water	Rubbed on affected area three times a day
							
				R	Body ache due to witchcraft			Crushed , mixed in cold water	Rubbed on skin cuts once a day

Ranunculaceae	*Clematis hirusta *Guill. & Perr. AA-05-07	Anthill on Open grassland	H	R	Swelling	4	14	Crushed	Massage affected area
							
				R	STI			Crushed , mixed in cold water	Extract drunk two glass thrice a day
							
				R	Cough			Crushed , boiled	Extract drunk twice a day
							
				Fl	Flu			Crushed	Inhaled

Rubiaceae	*Sarcocephalus latifolius *(SM.) E.A. Bruce AA-51-07	Grassland	SH	R	Piles	8	12	Burnt together with millet husk	Direct smoke to anus
							
				R	Scrotal hernia			Crushed , mixed in cold water	Extract drunk 20 ml once a day for a month
							
				R	Cough, stomachache			Crushed , boiled	Extract drunk 200 ml once a day
							
				R	STDs, worms			Crushed , boiled	Extract drunk one glass twice a day
							
				R	Diarrhoea			Crushed , mixed in cold water	Extract drunk half glass thrice a day
							
				R	Dysentery			Crushed , mixed in cold water	Extract drunk 200 ml thrice a day
	
	*Vangueria apiculata *K. Schum AA-16-07	Forest edge		S	Swollen feet , body	1	1	Crushed , mixed in cold water	Extract drunk half glass three times a day

Sapotaceae	*Vitallaria paradoxum *(C.F. Gaertn) Hepper AA-14-07	Wooded grassland	T	B	Diarrhoea	1	3	Dried , powder mixed in water	Drunk 20 ml two times a day

Simaroubaceae	*Harrisonia occidentalist *(Eng) L.AA-15-07	Ant hills	SH	R	Worms	2	2	Crushed , mixed in warm water	Extract drunk 500 ml a day
							
				L	Sores on head of children			Crushed	Rubbed on affected area twice a day

Solanaceae	*Capsicum frutescens *Rodsch. AA-13-07	Under big trees	SH	S	Backache	1	1	Crushed	Crushed bark rubbed on skin cuts
	
	*Solanum *sp AA-10-07	Ant hills , open grassland	SH	R	STI	5	2	Crushed , mixed in cold water	Extract drunk
							
				R	Ear disease			Crushed , mixed in cold water	Extract dropped in ear thrice a day
							
				R	Epilepsy			Crushed , mixed in cold water	
							
				R	Diarrhoea			Crushed , mixed in cold water	Extract drunk two teaspoon twice a day
							
				R	Headache			Crushed , mixed in cold water	Extract drunk
	
	*Solanum aculeatissimum *Jacq AA-28-07	Homestead	SH	R	Witchcraft	5	5	Crushed	Rub on skin cuts
							
				R	Hydrocelle			Crushed , mixed in cold water	Extract drunk
							
				R/F	snake bite			Crushed , mixed in cold water	Extract drunk
							
				F	Bone , muscle inflammation			Crushed , mixed in cold water*	Extract drunk

Tiliaceae	Grewia mollis Juss. AA-70-07	Open grassland	T	R	Swollen body part	1	1	Scraped	Plastered on swelling

Tricholomataceae	*Termitomyces microcarpus *AA-71-07	Forest		R	Boils	1	1	Crushed	Smeared on affected area

Umbellifereae	*Steganofaenia oraliacea *AA-63-07	Open grassland	SH	R	Measles	2	2	Crushed	Rubbed all over skin
							
				R	Swollen body			Dried , powdered	Added in one glass of water, drunk twice a day

Urticaceae	*Urtica massaica *Mildbr. AA-08-07	Forest, swamp	SH	L	Headache	4	4	Crushed	Rubbed on forehead
						
				R	Menstrual pain			Crushed	Extract drunk four teaspoon twice a day
							
				R	Boils			Crushed	Extract smeared on affected area once
							
				R	Cough			Crushed , mixed in cold water	Extract drunk

Verbenaceae	*Lanatana camara *L. AA-03-07	Garden edge, roadside	SH	L	Ringworms	4	5	Dried , powdered	Smeared on affected area
							
				L	Cataracts			Crushed , mixed in cold water	Extract dropped in eye
							
				R	snake bite			Crushed , mixed in cold water	Extract drunk 250 ml
							
				R	Epilepsy			Crushed , mixed in cold water	Extract drunk
	
	*Clerodendrum umbellatum *Poir AA-06-07	Gardens	SH	R	Cough	3	28	Crushed , boiled	Extract drunk third a glass
							
				L	Poison			Crushed , mixed in cold water	Extract drunk
							
				L	Abdominal pain			picked , cleaned	Chewed

Vitaceae	*Cyphostemma adenocaule *Descoings. ex Wild & R.B.Drumm. AA-01-07	Open grassland	C	R	Wounds	4	3	Sap collected	Rubbed on skin cuts
							
				R	Abortion			Crushed , mixed in cold water	Extract drunk three teaspoon three times a day
							
				R	Boils			Crushed	Extract smeared on affected area once
							
				R	Cough			Crushed , mixed in cold water	Extract drunk

Zingiberaceae	*Zingiber officinale *Roscoe AA-34-07	Homestead	H	R	Meningitis	2	4	Crushed	Rubbed on skin cuts once
							
				R	Cough			Crushed, warm water added	Drunk
	
	*Aframomum angustifolium *K. Schum AA-39-07	Open wooded grassland		S	Cholera	2	11	Crushed , mixed in cold water	Drunk
							
				S	Diarrhoea			Crushed , mixed in warm water	Drunk 50 ml two times a day for four days

**Key**: Plant Habit: SH-Shrub T-Tree C- Climber H-Herb G-Grass

Plant part used: R-Roots L-Leaves B-Bark S-Seeds F-Fruit S-Stem F-Flower

Mode of preparation: (1*2*3*) - Used in combination with other plant species

4x- Mixed in oil

These plant species were mainly obtained from open grassland area (41%), garden or farms (21%), homestead (13%) wooded grassland 11%, forest (7%) and least number was obtained from swamps (4%) and forest edge (3%).

The most commonly mentioned plant species by respondents were *Clerodendrum umbellatum *Poir (25%) *Securidaca longipedunculata *Fres. (17%) while the least mentioned among respondents includes; *Crotalaria ochroleuca *G.Don, *Albizia coriaria *Welw (0.9%). Fifty five percent of the plant species mentioned were used to treat more than one disease and 45% to treat only one disease.

A total of plant species documented, 25% were edible and formed part of local diet (Table 2). Fifty five percent of these were used in the treatment of more than one disease while 45% were believed to treat only one particular disease. The conservation status of the medicinal plant species is such that only 10% were cultivated and 90% were collected from the wild (Table 2).

**Figure 2:**
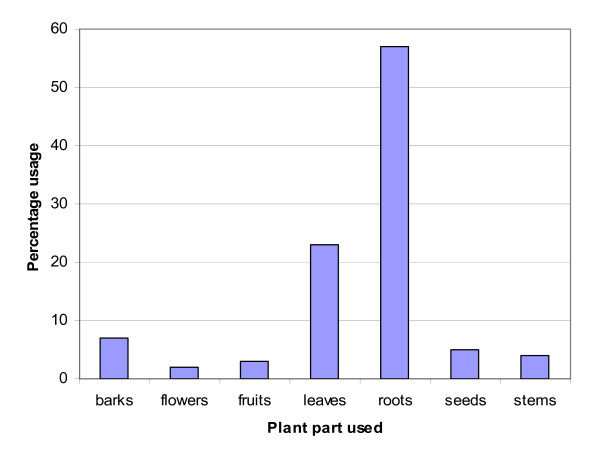
Different plant parts used for medicinal purpose and their percentages

Roots were the commonest plant parts (57%) being used; followed by leaves (23%) (Figure 2). The most underutilized plant part were found to be flowers with only 2% usage, fruits making up 3% and the rest of plant parts harvested making up 4%, 5% and 7% of stems, seeds and bark respectively.

Records reveal that a total of 41 conditions were treated with medicinal plant parts in Otwal and Ngai sub-counties in Oyam District. The common condition being treated in Ngai and Otwal sub counties was found to be abdominal pains and this was reported by 11% of the respondents, followed by cough at 10%. Other conditions such as wounds had 5.6% headache; epilepsy and STD/STI at 4.6%. Those least mentioned at below 1% were impotence, toothache, cholera, fever among others.

The most common way of preparing these medicinal plant species was mainly by crushing and extracting using cold water making up an overall 48%. This was followed by crushing plant parts and applied in that form at 20%. The least mode was found to be burning, and adding the ashes into bath water making up less that 1%.

On administration, oral administration through drinking was found to be the most frequently used at 69% and the least were through bathing with, massaging and smoking at less than 1%.

The main sources of indigenous knowledge of medicinal plant species were parents at 40%, grandparents at 35% (Table 3). The least sources of information about medicinal plant species were through dreams at 3.8% and in-laws 2.9%.

**Table 3:** Source of knowledge on medicinal plant species among the people of Ngai and Otwal sub counties in Oyam District.

Source of information	Frequency	Percentage
Parents	42	40
Peers	5	4.8
Grandparents	37	35.6
Traditional healers	13	13
In laws	3	2.9
Dreams	4	3.8
**Total**	**104**	**100.1**

The use of medicinal plant species was found to be driven mainly by its perceived effectiveness (34%), poverty, medical facilities being far (23%) and lack of medicines in hospitals (5%) (Table 4). The least use of medicinal plant species was due to referral from medical personnel (3%).

**Table 4:** Showing why medicinal plant species are in use

Reason for use	Frequency	%
Medical facilities far	35	23
Poverty	35	23
Conventional medicine don't work	7	5
Medicinal plant species effective	51	34
Hospitals lack medicines	7	5
Advice from medical workers	4	3
Easy to access	12	8

## Discussion

The 71 medicinal plant species of cultivated and wild types were greatly utilized by people of Oyam District as herbal remedies. These plant species fall under 42 families, with the family Asteraceae having the highest number of medicinal plant species. The family Asteraceae was also recorded as having the most number of medicinal plant species as other studies in other areas also reveals [15,16]. *Clerodendrum umbellatum, Securidaca longipedunculata, Clematis hirsuta *and *Conyza sumaternsis *were among the most frequently utilized species. The frequency of mention of a given plant species could be an indication of the prevalence of a given condition it can treat and its therapeutic values.

Roots were the most commonly harvested plant part of the medicinal plants compared to any other part. This form of harvesting however, is threatening to the survival of the plant. Plant species such as *Lantana camara, Urtica massaica *had leaves and roots being harvested. Harvesting of two or more plant parts can be more damaging especially when the roots and barks/stem are harvested. Thus from the conservation point of view, the high utilization of roots of plant species in Oyam District put these plant species at a risk because of the damages inflicted on the plant species. This was also noted in other areas [6].

Many of these plant species treated more than one condition and are being used in combination. This pattern of using medicinal plant species for varying conditions was also observed among the local communities in Mabira Forest Reserve area [15]. However, it was found that locals usually mix the medicinal plant species to ensure effectiveness in treating a given ailment [17,18]. This was also observed in Ngai Sub County, where the extent of knowledge of medicinal plant mixing determined the success of a traditional healer. Medicinal plant are strongly believed by the local people of Ngai and Otwal to be effective and this among other reasons explain why they have continued to use them, thus their reliance on them for basic healthcare. This trend was also observed among the people living around Queen Elizabeth National Park in western Uganda [3,6]

Abdominal pain and cough were the most frequently treated ailments. These are diseases associated with personal hygiene. The study area has had IDP camps which was always associated with poor hygiene and over-crowding. The high frequency of mention of these diseases were directly associated with the high prevalence of these diseases in the area. This goes on to explain why many of the medicinal plant species mentioned were used for treating these ailments indicating widespread knowledge of medicinal plant species used for their treatment. For example, 25% of respondents mentioned that *Clerodendrum umbellatum *was used for treatment of abdominal pain.

The most common method of preparation of medicinal plant species before being administered was found to be applied to most plant species. This involved crushing and extracting plant materials using cold/warm water and boiling. Those that were boiled were effectively extracted compared to use of cold water, since boiling also preserves the medicine longer. Oral administration was noted as number one mode of dispensing of herbal medicine. This mode of administration of herbal medicine was also reported elsewhere [6,18].

Some of these plant species are popular and used all over Uganda and are on sale in most markets. For instance *Cleome gynandra, Cajanus cajan*, *Vitallaria paradoxum*, *Capsicum frutescens *were found to be sources of food and were being eaten not only locally but also nationally and internationally [3].

Some studies carried out in and outside Uganda showed that some of these plant species were potent as medicine. A plant like *Aspilia africana*, is said to have high antiplasmodial activity [19]. Some other plant species mentioned elsewhere as medicine include *Cassia occidentalis *which is used in Burkina Faso as stimulant [3].

### Conservation issues

It should be noted that a high percentage of these plant species are harvested from the wild, but with no consideration for domestication hence threatening their existence. The plant species are being overexploited, and the rapid environmental degradation coupled with insurgency has put mounting pressure on the environment. This may lead to the disappearance of many species of medicinal plants of economic value. According to one of the local traditional practitioners, Okello Okiko, the use of medicinal plant species is becoming expensive since some of the plant species are hard to find and one has to risk going to restricted conservation areas to get the plant species. Since the knowledge comes at a price, many people are even too poor to pay for the herbalist services, hence a reduction in number of clients.

The disappearance of medicinal plant species can also be attributed to over use, agricultural activities and insecurity. Domestication of medicinal plant species is probably not taken seriously. Some medicinal plant species which have been proved potent have been over used [4,20]. The mode of harvesting which involves the use of roots also posed a threat to the existence of these plant species. In most of the plant species, their roots were being used.

## Conclusions

The 71 medicinal plant species of cultivated and wild types were greatly utilized for treating a total of 41 different ailments by people of Ngai and Otwal Sub Counties. Thirty nine percent of the recorded plant species were reported for treating stomach related ailments. The most commonly mentioned plant species by respondents were *Clerodendrum umbellatum *Poir (25%). Of the total of plant species documented, 25% were edible and formed part of local diet. The main sources of indigenous knowledge of medicinal plant species were parents at 40%.

Roots were the most commonly harvested plant part of the medicinal plant species compared to any other part. The most common method of preparation of medicinal plant species before being administered was found to be applied to most plant species. However, it was noted that some of these medicinal plant species are disappearing very first. The disappearance of medicinal plant species can be attributed to over use, agricultural activities and insecurity. Domestication of medicinal plant species is probably not taken seriously.

The use of medicinal plant species in primary health care is still a common practice in Ngai and Otwal Sub-County. The inadequate health services and abject poverty still make these people dependent on herbal medicine for their day to day health needs.

The generation gap caused by the over 20 years of insurgency in the area has brought about knowledge gap between the young and the old with regard to medicinal plant species.

## Recommendations

• There is need for ex-situ conservation of the useful medicinal plant species

• There is need for community awareness and education concerning the values of medicinal plant species of the area especially among the young people.

• Further studies should be done on the medicinal plant species to determine their pharmacological potentials.

• Government should develop policy to integrate use of medicinal plant species in health care at national level

## Competing interests

The authors declare that they have no competing interests.

## Authors' contributions

AA identified the research area and title, collected field data, carried out statistical analysis and drafted the manuscript. MMK and OOH participated in refining the title, formulation of the research problem, data analysis and drafting as well as enrichment of the manuscript. All authors read and approved the final manuscript.
